# The Bulbocavernosus Reflex in the Differential Diagnosis of Multiple System Atrophy with Predominant Parkinsonism and Parkinson’s Disease

**DOI:** 10.3389/fneur.2017.00697

**Published:** 2018-01-05

**Authors:** Huan-jie Huang, Xing-yu Zhu, Xun Wang, Zhang-yang Wang, Wan-li Zhang, Bi-cheng Chen, Chun-feng Liu

**Affiliations:** ^1^Department of Neurology, Suzhou Clinical Research Center of Neurological Disease, The Second Affiliated Hospital of Soochow University, Suzhou, China; ^2^Department of Neurology, The First Affiliated Hospital, Wenzhou Medical University, Wenzhou, China; ^3^School of the First Clinical Medical Sciences, Wenzhou Medical University, Wenzhou, China; ^4^Department of Neurology, Huashan Hospital, Shanghai Medical College, Fudan University, Shanghai, China; ^5^Zhejiang Provincial Top Key Discipline in Surgery, Wenzhou Key Laboratory of Surgery, Department of Surgery, The First Affiliated Hospital, Wenzhou Medical University, Wenzhou, China

**Keywords:** multiple system atrophy, Parkinson’s disease, bulbocavernosus reflex, electrophysiological test, differential diagnosis

## Abstract

Multiple system atrophy with predominant parkinsonism (MSA-P) is a degenerative disorder that presents with autonomic dysfunction, atypical parkinsonism, and ataxia. Parkinson’s disease (PD) is an age-related neurological disorder of the central nervous system. Differentiation between MSA-P and PD is important because treatments, complications, and prognoses differ. The bulbocavernosus reflex (BCR) tests the afferent and efferent signals of the pudendal nerve as well as the sacral cord. In this study, we investigated differences in BCR parameters between MSA-P and PD patients. Thirty-eight MSA-P patients and 32 PD patients were selected to participate in our electrophysiological investigations. The Keypoint EMG/EP system was used to induce the BCR, and latencies and amplitudes were recorded for systematic statistical analyses. Area under the curve of the receiver operating characteristic was used to assess the specificity and sensitivity of the BCR parameters. A BCR was elicited in 76.32% of MSA-P patients and 93.75% of PD patients. The BCR latencies of the MSA-P group were longer than those of the PD group (*p* < 0.001). In addition, the MSA-P group had a lower BCR amplitude compared to the PD and control groups (*p* < 0.001). We discovered the difference between MSA-P and PD through BCR latencies and amplitudes. Compared to PD patients, MSA-P patients have longer latencies and lower amplitudes. Therefore, the BCR may be used to discriminate between MSA-P and PD in some cases.

## Introduction

The differentiation between Parkinson’s disease (PD) and other parkinsonian diseases is a difficult issue in clinical diagnosis (1). The symptoms of multiple system atrophy with predominant parkinsonism (MSA-P) and PD are superimposed and similar. Ten percent of probable PD patients are eventually diagnosed with MSA (2). PD predominantly affects the motor system and sometimes influences the autonomic nervous system; however, MSA-P mainly affects the autonomic nervous system. When the autonomic nervous system is altered, constipation, bladder problems, and orthostatic hypotension often occur (3, 4). The parkinsonism paired with an autonomic nervous system disorder makes it hard to differentiate between MSA-P and PD patients (5). Different methods have been used to help make this discrimination and, of these, imaging methods have been the most popular (6). Other methods based on autonomic nervous system disorders have also been studied (7). Despite these previous attempts, an objective examination that includes the autonomic nervous system and that renders a greater possibility for differential diagnosis is still needed.

The bulbocavernosus reflex (BCR) causes the contraction of the bulbocavernosus muscle by stimulating the pudendal nerve, and this contraction can be recorded using the electromyogram technique. The BCR tests the afferent and efferent signals of the pudendal nerve as well as the sacral cord (8). A lesion of the reflex arc influences the BCR latencies and amplitudes. Over the last 20 years, the BCR has been used in the diagnosis of various neurogenic diseases, including neurogenic impotence, spinal shock, and cauda equine syndrome (9–11). The pudendal nerve somatosensory-evoked potential responses (PSEP) share the same pathway with the BCR before entering the spinal cord. Therefore, PSEP together with the BCR could be used to locate a lesion in the pathway. Previous research from our group suggests that an abnormality of BCR exists in MSA patients (12). Given that MSA and PD share the same symptoms of autonomic nervous system disorders, the abnormality may also exist in PD patients. In this study, we intend to distinguish between MSA-P and PD patients using the BCR measurement. Specifically, our study compared the latencies and amplitudes of the BCR and PSEP in 38 probable MSA-P patients, 32 PD patients, and 30 normal, healthy subjects. Statistical analyses were performed to differentiate between MSA-P and PD.

## Materials and Methods

### Subject

In this study, 38 probable MSA-P patients and 32 PD patients were selected to participate in the electrophysiological investigations between December 2012 and October 2014. All of the patients were referred to the First Affiliated Hospital of Wenzhou Medical University. All MSA-P patients met the inclusion criteria published in 2008 (13). Probable MSA-P patients were diagnosed by two inclusion criteria: autonomic failure involving urinary incontinence (inability to control the release of urine from the bladder, with erectile dysfunction in males) or an orthostatic decrease of blood pressure and poorly levodopa-responsive parkinsonism (bradykinesia with rigidity, tremor, or postural instability). Likewise, all PD patients met the Movement Disorder Society’s clinical diagnostic criteria (14). PD patients were characterized by tremor at rest, rigidity, akinesia, and postural instability. In addition, all patients were evaluated under magnetic resonance imaging (MRI) and a follow-up visit. We also recruited 30 healthy subjects with no history of neurological or psychiatric diseases as a control group. All healthy controls underwent thorough clinical and neurologic examinations. Subjects with a history of parkinsonism, ataxia, autonomic dysfunction, or neurologic diseases (e.g., epilepsy or stroke) were excluded. Moreover, male subjects with prostatic diseases were excluded. MRI was used to exclude those subjects with cerebrovascular or space-occupying lesions.

All participants gave their written informed consent in accordance with the Declaration of Helsinki and agreed to participate in the study. This study was conducted under the approval of the Ethical Decision Committee of the Research Administration at the First Affiliated Hospital of Wenzhou Medical University (CR2009041).

### Electrophysiology

The Keypoint EMG/EP system (Dantec, Bristol, UK) was used to conduct the BCR and PSEP tests. A ground electrode was placed against the thigh of patients in the lithotomy position. The temperature of the skin surface was maintained above 32°C.

### Bulbocavernosus Reflex

In males, ring-shaped stimulating electrodes were placed on the body or at the root of the penis. The positive pole was located at the corona glandis. In females, clamp-stimulating electrodes were placed near the pubic symphysis. The positive and negative poles were put on the clitoris and the labia vulvae, respectively. The BCR was recorded by concentric needle electrodes inserted into the scrotum at the perianal region near the midline. The stimulus intensity was seven times the individual’s sensory threshold, and the electrode impedance was <5 kΩ. Stimulation of the square wave was 1.9 pulses/s, and we recorded the average value of 20 reflection waves. The scanning time was 5 ms/division, bandwidth range was 10 Hz to 2 kHz, and persistence time was 100 ms. Latencies were calculated based on the beginning of the stimulus and the start of reflex response.

### Pudendal Nerve Somatosensory-Evoked Potential Responses

The electrodes used to evoke pudendal nerve potentials were identical to those used to evoke the BCR, and they were applied to the same places. However, the intensity of the stimulus was three times the individual’s sensory threshold. Similarly, the electrode impedance was maintained at 5 kΩ. The frequency of the square wave was 5 pulses/s and was averaged across 200 waves. The scanning time was set to 0.2 ms/division, and the relevant persistence time was 100 ms, with a bandwidth ranging from 10 Hz to 5 kHz. The recording electrode was located 2 cm behind Cz, and the reference electrode was placed on the forehead at Fpz. The PSEP P41 wave was detected after stimulus delivery.

### Statistical Analysis

The data were analyzed using the Statistical Package for the Social Sciences 22.0 (IBM, CA, USA) and Stata SE 12 (StataCorp, LP, USA). Normal distribution and homogeneity of variance were identified using the Kolmogorov–Smirnov test and Shapiro–Wilk test. A chi-square test was used to compare the elicitation rates of BCRs between the MSA-P, PD, and control groups. A one-way analysis of variance (ANOVA) was used to analyze the differences in the BCR parameters between the three groups (MSA-P, PD, and control) in males. PSEP parameters also analyzed with an ANOVA. A Bonferroni multiple comparison test was applied when significant differences were identified. This test revealed further contrasts between the groups. A Kruskal–Wallis rank test was used to compare the BCR and PSEP parameters between the three (MSA-P, PD, and control) in females. A Nemenyi test was applied to further evaluate the differences between each group. A receiver operating characteristic (ROC) analysis was used to evaluate the specificity and sensitivity of the BCR parameters. A value of *p* ≤ 0.05 was considered statistically significant.

## Results

### General Patient Characteristics

Thirty-eight probable MSA-P patients and 32 PD patients participated in the study. The age of the probable MSA-P patients ranged from 36 to 74 years, and the mean age was 54.21 ± 7.92 years. 39% of patients (15 cases) had erectile dysfunction, 45% of patients (17 cases) had urinary incontinence, and 76% of patients (22 cases) had orthostatic hypotension. In addition, the age of the diagnosed PD patients ranged from 35 to 77 years, and the mean age was 55.23 ± 8.75 years. 22% of patients (7 cases) had erectile dysfunction, 3% of patients (1 case) had urinary incontinence, and 16% of patients (5 cases) had orthostatic hypotension. Both the MSA-P and PD groups suffered from autonomic failure (see Table 1).

**Table 1:** General characteristics and elicitation rates of the bulbocavernosus reflex (BCR) latencies between multiple system atrophy with predominant parkinsonism (MSA-P), Parkinson’s disease (PD), and control groups.

		MSA-P	PD	Control
Ages	Mean	54.21 ± 7.92	55.23 ± 8.75	57.16 ± 7.25
	Ranges	36–74	35–77	38–70
Males		18	17	14
Females		20	15	16
Total		38	32	30
	Duration (month)	26.4	46.5	–
	Range	4–72	6–240	–
Erectile dysfunction		15	7	–
Urinary incontinence		17	1	–
Orthostatic hypotension		22	5	–
Available for BCR responses		29	30	30
Elicitation rates of BCR^a^		76.32%	93.75%	100%

*^a^χ^2^ = 10.01, p = 0.003*.

### BCR Measurements

**Figure 1:**
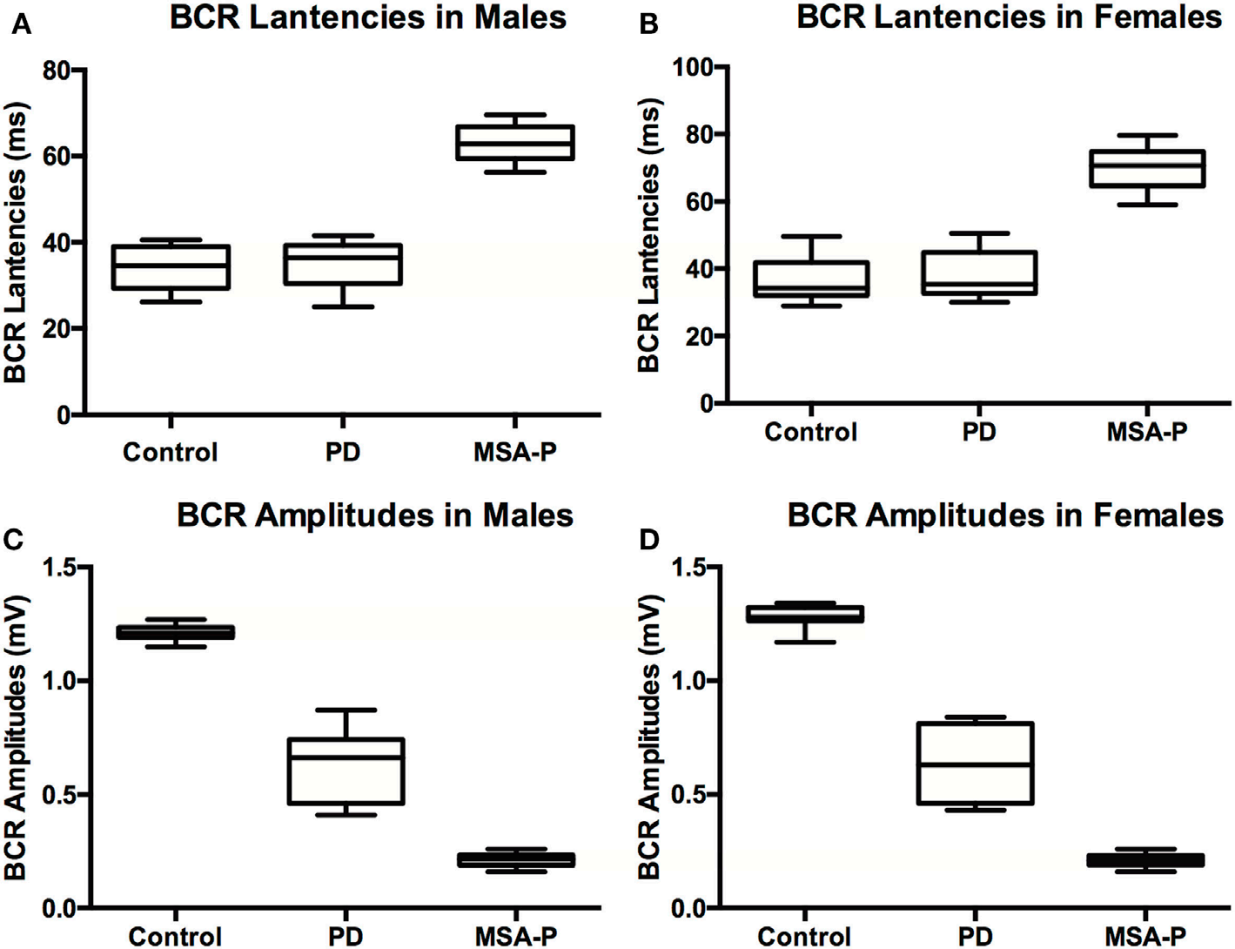
Bulbocavernosus reflex (BCR) latencies and amplitudes between multiple system atrophy with predominant parkinsonism (MSA-P), Parkinson’s disease (PD), and control groups in males **(A)** and females **(B)**. BCR latencies were not significantly different between control and PD patients, but MSA patients had prolonged latencies compared to the other groups in males **(C)** and females **(D)**. BCR amplitudes were significantly lower in MSA-P patients than those in the control group, and amplitudes of PD patients fell in between control and MSA-P amplitudes.

Elicitation rates in the MSA-P, PD, and control groups were 76.3% (29/38), 93.75% (30/32), and 100% (30/30). There was a significant difference in the BCR elicitation rates between the three groups (χ^2^ =10.01, *p* = 0.003). The BCR latencies in the MSA-P group were longer than those of the control and PD groups. There was no significant difference between the PD and control group latencies in either gender (*p* = 1.000 in male, *p* = 0.806 left, *p* = 0.902 right in female, see Table 2; Figures 1A,B). The BCR amplitudes of the MSA-P, PD, and control groups were significantly different from each other (*p* < 0.01, see Table 3; Figures 1C,D). The MSA-P group had the lowest amplitudes, and the control group had the highest amplitudes. In ROC curve, significant differences were found between the groups (area under the curve = 1.000).

**Table 2:** Comparison of the mean or median bulbocavernosus reflex latencies between multiple system atrophy with predominant parkinsonism (MSA-P), Parkinson’s disease (PD), and control groups.

	Latencies of males (ms)		Latencies of females (ms)	
	Left	Right	Left	Right
MSA-P	62.843 ± 1.136	63.221 ± 1.172	69.647 ± 1.461	69.827 ± 1.461
PD	34.780 ± 5.234	34.793 ± 4.939	37.600 ± 6.255	37.393 ± 6.215
Control	33.800 ± 5.022	34.243 ± 5.142	36.313 ± 6.048	36.475 ± 6.180
	*F* = 162.613	*F* = 166.245	*H* = 30.122	*H* = 29.893
	*p* < 0.001	*p* < 0.001	*p* < 0.001	*p* < 0.001

*Male*.

*MSA-P vs Control, PD vs MSA-P p < 0.001, PD vs Control p = 1.000*.

*Female (*Left*, Nemeyi test)*.

*PD vs Control p = 0.806, MSA-P vs Control, MSA-P vs PD p < 0.001*.

*Female (*Right*, Nemeyi test)*.

*PD vs Control p = 0.902, MSA-P vs Control, MSA-P vs PD p < 0.001*.

**Table 3:** Comparison of the mean or median bulbocavernosus reflex amplitudes between multiple system atrophy with predominant parkinsonism (MSA-P), Parkinson’s disease (PD), and control groups.

	Amplitudes of males (mV)		Amplitudes of females (mV)	
	Left	Right	Left	Right
MSA-P	0.209 ± 0.008	0.207 ± 0.007	0.207 ± 0.008	0.205 ± 0.009
PD	0.631 ± 0.156	0.625 ± 0.152	0.631 ± 0.156	0.625 ± 0.152
Control	1.213 ± 0.033	1.210 ± 0.032	1.286 ± 0.046	1.272 ± 0.042
	*F* = 387.214	*F* = 412.274	*H* = 40.047	*H* = 40.064
	*p* < 0.001	*p* < 0.001	*p* < 0.001	*p* < 0.001

*Male*.

*MSA-P vs Control, PD vs Control, PD vs MSA-P, p < 0.001*.

*Female (*Left and Right*, Nemeyi test)*.

*PD vs Control p = 0.006, MSA-P vs Control p = 0.009, MSA-P vs PD p < 0.001*.

### PSEP Measurements

No significant differences existed between the MSA-P, PD, and control groups in either the males or females (see Table 4).

**Table 4:** Comparison of the mean pudendal nerve somatosensory-evoked potential responses P41 latencies and amplitudes between multiple system atrophy with predominant parkinsonism (MSA-P), Parkinson’s disease (PD), and control groups.

	Males		Females	
	Latencies (ms)	Amplitudes (mV)	Latencies (ms)	Amplitudes (mV)
MSA-P	42.911 ± 0.659	2.684 ± 0.065	42.067 ± 0.643	2.626 ± 0.083
PD	41.480 ± 2.944	2.765 ± 0.286	43.087 ± 2.986	2.672 ± 0.394
Control	40.629 ± 1.931	2.650 ± 0.330	44.238 ± 2.256	2.654 ± 0.344
	*H* = 5.308	*H* = 0.595	*H* = 5.695	*H* = 0.218
	*p* = 0.070	*p* = 0.556	*p* = 0.058	*p* = 0.897

## Discussion

In this study, we discovered that a difference found in BCR parameters could help to distinguish between MSA-P and PD. MSA-P patients exhibited a lower BCR elicitation rate than did PD patients (76.3 vs 90.9%). The BCR parameters revealed differences between the MSA-P, PD, and control groups. MSA-P patients exhibited longer latencies and lower amplitudes than did the PD group. In addition, both of these parameters could significantly distinguish MSA-P using an ROC curve.

The afferent somatic sensory pathway of the BCR includes the pudendal nerve. The efferent somatic motor pathway is composed of the spinal segments S2–S4, including Onuf’s nucleus and the pudendal nerve. An impulse contracts the bulbocavernosus muscle (8, 15). The afferent somatic sensory pathway of PSEPs comprises two segments, and the anterior segment shares the same afferent pathway with the BCR. An impulse is then transmitted to the cerebral cortex through the spinal cord. The combination of the BCR and PSEPs could be used to locate a lesion in some neuropathies, such as diabetic neurogenic bladder and cauda equine syndrome.

In this study, MSA-P and PD patients had an abnormal BCR and normal PSEPs. The elicitation rates of MSA-P patients were lower than those of PD patients. In addition, we failed to elicit the BCR in 23.68% of the MSA-P patients. The lesion of reflex arc in the BCR of the MSA-P patients was more serious than in the PD patients. Furthermore, MSA-P patients exhibited lower BCR amplitudes than did PD patients. However, the PSEPs revealed no differences between the MSA-P, PD, and control subjects. The PSEP data cannot be used to distinguish between MSA-P and PD patients in our study. The pathway of PSEPs seemed to be complete in both the MSA-P and PD patients. As the BCR and PSEPs share the same afferent pathway, the lesions are likely to be located on the efferent pathway, which consists of the spinal segments S2–S4, including Onuf’s nucleus, or on the pudendal nerve. These findings support the results of several previous studies. Konno et al. (16) found deprivation loss of somatic motor neurons in Onuf’s nucleus. Moreover, the previous studies of Ref. (17) have found that the external anal sphincter electromyography (EAS-EMG) shares the same pathway with BCR. In addition, their results also show the degeneration of Onuf’s nucleus anterior horn cell. According to these studies, the lesion in the BCR is likely positioned in Onuf’s nucleus. It is also important to note that compared to the EAS-EMG, the methods used in our study are non-invasive.

Parkinson’s disease is characterized by motor symptoms, such as resting tremor, rigidity, and slowness of movement, which can all be explained by the degeneration of neurons in the central nervous system. However, the non-motor-related symptoms in PD patients, such as constipation, bladder problems, and orthostatic hypotension have recently gained increasing attention. In this study, we found that the BCR amplitudes were lower than those in the control group, but higher than those in the MSA-P group. This suggests that certain lesions most likely involve the BCR in PD patients, but this condition is more serious in MSA-P patients. The autonomic nervous system is more seriously affected in MSA-P patients.

Shared non-motor symptoms in PD and MSA-P patients make it difficult to differentially diagnose MSA-P and PD patients, especially at an early stage. Multiple tests have been used in the last few decades in an attempt to solve this problem. Imaging methods, such as positron emission tomography, diffusion weighted image, and diffusion tensor image, have been used previously (18–20). The application of MRI does improve clinical accuracy (6, 21). Moreover, 3T susceptibility-weighted imaging is more susceptible to putaminal changes and lesion asymmetry (21). Using brain volumetry with MRI is inefficient sometimes (22). In mildly symptomatic PD and MSA-P patients, brain perfusion is altered through the single-photon emission computed tomography method (23). However, with this technique, some influencing factor could disturb the results, and the process of data analysis is complex and time-consuming. In addition, the cost of radiological examination should also be considered, especially in developing countries. Other methods, such as measuring cerebrospinal fluid levels lack accuracy (24, 25). The examination of autonomic nerve function has become a research focus in recent years (7, 26, 27). This method consists of a battery of cardiovascular autonomic tests, including orthostatic hypotension, systolic and diastolic blood pressure, and other tests. Using this method, Pilleri et al. (28) found that MSA patients exhibited increased nocturnal heart rate and reduced nocturnal decline of heart rate compared to PD patients. Pros and cons exist in the methods above; no single test can have both favorable sensitivity and specificity. An objective examination renders a greater possibility for accurate differential diagnosis.

Our study has some limitations. For example, the sample population of our study was restricted to available patients. In addition, all patients with MSA-P and PD were diagnosed clinically rather than pathologically.

In conclusion, we observed a difference between MSA-P and PD patients in the BCR. Moreover, we consider the BCR test to be a non-invasive, convincing, objective, and economic approach to discriminate between MSA-P and PD patients. Supplementary study with more cases will validate our findings. Further application including the trace of BCR alteration with the progression of MSA-P or the association between BCR parameters and prognosis may help us better monitor these devastating neurological diseases.

## Ethics Statement

All participants gave their written informed consent in accordance with the Declaration of Helsinki and agreed to participate in the study. This study was conducted under the approval of the Ethical Decision Committee of the Research Administration at the First Affiliated Hospital of Wenzhou Medical University (CR2009041).

## Author Contributions

H-jH and X-yZ analyzed the data, wrote and revised the article. Z-yW participated in the writing and revised the article. XW and W-lZ performed the clinical tests and collected the data. B-cC and C-fL conceived and designed the study and obtained ethics approval.

## Conflict of Interest Statement

The authors declare that the research was conducted in the absence of any commercial or financial relationships that could be construed as a potential conflict of interest.
